# Implications of Angiogenesis Involvement in Arthritis

**DOI:** 10.3390/ijms19072012

**Published:** 2018-07-10

**Authors:** Iona J. MacDonald, Shan-Chi Liu, Chen-Ming Su, Yu-Han Wang, Chun-Hao Tsai, Chih-Hsin Tang

**Affiliations:** 1 Graduate Institute of Basic Medical Science, China Medical University, Taichung 40402, Taiwan; ionamac@gmail.com (I.J.M.); sdsaw.tw@yahoo.com.tw (S.-C.L.); 2 Department of Orthopedic Surgery, China Medical University Hospital, Taichung 40447, Taiwan; ritsai8615@gmail.com; 3 Department of Biomedical Sciences Laboratory, Wenzhou Medical University, Dongyang 325035, Zhejiang, China; proof814@gmail.com; 4 Graduate Institute of Biomedical Science, China Medical University, Taichung 40402, Taiwan; laecy0313@gmail.com; 5 School of Medicine, China Medical University, Taichung 40402, Taiwan; 6 Chinese Medicine Research Center, China Medical University, Taichung 40402, Taiwan; 7 Department of Biotechnology, College of Health Science, Asia University, Taichung 41354, Taiwan

**Keywords:** rheumatoid arthritis, osteoarthritis, angiogenesis, cytokines, chemokines

## Abstract

Angiogenesis, the growth of new blood vessels, is essential in the pathogenesis of joint inflammatory disorders such as rheumatoid arthritis (RA) and osteoarthritis (OA), facilitating the invasion of inflammatory cells and increase in local pain receptors that contribute to structural damage and pain. The angiogenic process is perpetuated by various mediators such as growth factors, primarily vascular endothelial growth factor (VEGF) and hypoxia-inducible factors (HIFs), as well as proinflammatory cytokines, various chemokines, matrix components, cell adhesion molecules, proteases, and others. Despite the development of potent, well-tolerated nonbiologic (conventional) and biologic disease-modifying agents that have greatly improved outcomes for patients with RA, many remain resistant to these therapies, are only partial responders, or cannot tolerate biologics. The only approved therapies for OA include symptom-modifying agents, such as analgesics, non-steroidal anti-inflammatory drugs (NSAIDs), steroids, and hyaluronic acid. None of the available treatments slow the disease progression, restore the original structure or enable a return to function of the damaged joint. Moreover, a number of safety concerns surround current therapies for RA and OA. New treatments are needed that not only target inflamed joints and control articular inflammation in RA and OA, but also selectively inhibit synovial angiogenesis, while preventing healthy tissue damage. This narrative review of the literature in PubMed focuses on the evidence illustrating the therapeutic benefits of modulating angiogenic activity in experimental RA and OA. This evidence points to new treatment targets in these diseases.

## 1. Introduction

Angiogenesis, the formation of new capillaries from pre-existing vessels, is one of the earliest histopathologic findings in chronic, non-infectious arthritis and is a potential target for therapeutic intervention. The most common forms of chronic, non-infectious arthritis are rheumatoid arthritis (RA) and osteoarthritis (OA). The development of potent, well-tolerated non-biologic (conventional), and biologic disease-modifying agents used alone and in combination to induce and maintain tight control of disease have greatly improved outcomes for patients with RA, but treatment resistance is common or patients achieve only partial responses [1]. Moreover, few treated RA patients achieve sustained remission, so require ongoing pharmacologic therapy [1]. For OA, the only approved therapies include symptom-modifying agents, such as analgesics, non-steroidal anti-inflammatory drugs (NSAIDs), steroids, and hyaluronic acid [2,3]. None of the available treatments slow the disease progression, or restore the original structure and function of the damaged joint [2,3]. Joint replacement surgery is considered to be the definitive treatment for alleviating pain and restoring function [3]. New treatments are needed that not only target inflamed joints and control articular inflammation in RA and OA, but also selectively inhibit angiogenesis, while preventing healthy tissue damage. This article discusses the current therapeutic situation in RA and OA, and focuses on future strategies that look very promising for the management of angiogenesis in these diseases. The literature consulted for this Review is ordered by topics discussed in Table 1 (RA) and Table 2 (OA).

## 2. Rheumatoid Arthritis

Conventional synthetic disease-modifying anti-rheumatic drugs (csDMARDs) such as methotrexate have long been the mainstay of treatment for RA [4]. However, these treatments fail to slow radiographic progression, with the exception of antimalarials, and safety issues have necessitated additional treatment strategies [4]. An improved understanding of the immunological pathway mediating RA has revealed that pro-inflammatory cytokines such as tumor necrosis factor alpha (TNF-α), interleukin (IL)-1, and IL-6 play a key role in the pathogenesis of RA [4,5]. The development of biologic DMARDs (bDMARDs) that target these cytokine pathways and mediators in the inflammatory cascade has improved outcomes for patients with RA. Compared with the traditional DMARDs, bDMARDs have a more rapid onset of action and are associated with sustained and clinically significant suppression of signs and symptoms, as well as inhibition of joint damage [4,6]. In general, their methods of action are also more directed, defined and targeted, compared with traditional DMARDs.

Together with bDMARD therapies, the introduction of early treatment initiation and the treat-to-target principle have helped to transform the management of RA and many patients achieve clinical remission early in the disease course, although this is not possible for all patients. The level of clinical response and efficacy of bDMARDs differ amongst individual patients [7]. An additional complication with bDMARD therapies is that they are typically given as injectable formulations, which poses a significant burden on health systems worldwide and may severely compromise treatment compliance where patients must travel long distances to access health centers [8]. The majority of patients with RA would prefer oral treatment to an injection or intravenous infusion [9]. Better personalized treatment algorithms are called for that achieve rapid remission in all patients, preventing disability, restoring and maintaining quality of life, without unwanted toxicity [8]. The more that is understood about the disease process in RA, the better.

**Figure 1:**
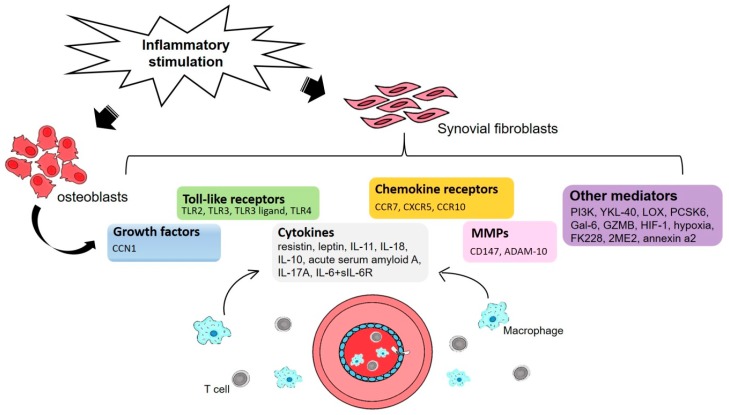
An illustration of the proinflammatory process underlying rheumatoid arthritis (RA) angiogenesis in synovial fluid. Inflammatory stimulation activates RA osteoblasts and synovial fibroblasts that in turn modulate the expression of growth factors, Toll-like receptors, chemokine receptors, cytokines, matrix metalloproteinases (MMPs) and other mediators that are involved at different stages of angiogenesis. Recruitment of macrophages and T cells from the blood into the inflammatory process ensure the maintenance and progression of angiogenesis.

Angiogenesis is essential for the expansion of synovial tissue in RA: pre-existing vessels facilitate the entry of blood-derived leukocytes into the synovial sublining, to generate and potentiate inflammation. Several steps are involved in angiogenesis, each of which is modulated by specific factors [10]. The process starts with growth factors such as vascular endothelial growth factor (VEGF) and fibroblast growth factor (FGF) binding to their cognate receptors on endothelial cells (ECs) and activation of these cells to produce proteolytic enzymes. Subsequently, the basement membrane is degraded by matrix metalloproteinases (MMPs), leading to migration and further endothelial proliferation to vascular tubules that are in part developed by adhesion molecules such as integrins. Lastly, blood vessels are stabilized by pro-angiogenic factors such as Ang1, followed by incorporation of pericytes into the newly formed basement membrane to facilitate the blood flow process (see Figure 1).

## 3. The Involvement of Toll-Like Receptors in RA Disease

Toll-like receptors (TLRs), a family of germline-encoded type I trans-membrane proteins, enable the innate immune system to recognize pathogen-associated molecular patterns [11]. High levels of TLR2, TLR3, TLR4, and TLR7 expression have been found in the RA synovium; TLR3 is highly expressed in fibroblast-like synoviocytes (FLS), while TLR2 and TLR4 expression is increased in the perivascular regions of the joint, at the sites of attachment and invasion into cartilage/bone, and on synovial macrophages [11,12]. In vitro and ex vivo studies have investigated the roles of TLR2 and TLR3 in RA pathogenesis.

Evidence indicates that the activation of TLR3 in RA FLS increases VEGF and IL-8 production and upregulates the genes for these proteins at the transcriptional level after stimulation of FLS with the TLR3 ligand, a polyinosinic-polycytidylic acid (poly(I:C)) [11]. Treatment with the nuclear factor-kappa B (NF-κB) inhibitors, pyrrolidine dithiocarbamate and parthenolide, abrogated the stimulatory effect of poly(I:C) on the production of VEGF and IL-8 in RA FLS, which suggests that targeting the NF-κB signaling pathway may prevent the upregulation of pro-angiogenic molecules in RA FLS [11].

Another study used RA whole-tissue synovial membrane explants to demonstrate that TLR2 activation induces angiogenic tube formation and angiopoietin-2 (Ang2) expression, EC invasion and migration, as well as increased MMP-2 and MMP-9 expression by RA synovial explants [12]. These effects were inhibited by Tie2 receptor blockade, suggesting that TLR2-induced angiogenic processes are in part mediated through the Tie2 pathway.

## 4. Vasohibin-1 mRNA Expression in RA Synovial Fibroblasts

In vitro investigations have shown that expression of vasohibin-1, a novel endothelium-derived VEGF-inducible angiogenesis inhibitor, correlates significantly with histological inflammation score (*r* = 0.842; *p* = 0.002) [13]. Those researchers also found that stimulation with VEGF induced the expression of vasohibin-1 mRNA in RA synovial fibroblasts (RASFs) under normoxic conditions, while stimulation with cytokines TNF-α and IL-1β induced vasohibin-1 mRNA expression under a hypoxic condition (1% O_2_).

Japanese researchers have suggested that histone deacetylase (HDAC) inhibitors may help to suppress angiogenesis-related factors in RA synovial tissue [14]. They stimulated RASFs with TNF-α and IL-1β then incubated them under hypoxic conditions (1% O_2_) with different concentrations of FK228, a specific HDAC inhibitor. FK228 dose-dependently down-regulated the expression of hypoxia-inducible factor-1 alpha (HIF-1α) and VEGF mRNA. FK228 also reduced the levels of HIF-1α and VEGF protein in the RASFs. Intravenous administration of FK228 (2.5 mg/kg) suppressed VEGF expression and inhibited angiogenesis in synovial tissue analyzed from mice with collagen-induced arthritis (CIA), a frequently used autoimmune animal model in the study of RA, as the signs of disease resemble features of human inflammatory arthritis and thus enable investigators to test hypothetical mechanisms of immune-mediated joint disease and examine the comparative efficacy of pending RA therapies during preclinical development.

The findings from the Japanese researchers are extended by in vivo research demonstrating anti-angiogenic and anti-proliferative activity with 2-methoxyestradiol (2ME2) in the rat CIA model [15]. In preventive protocols, 2ME2 significantly inhibited the onset and reduced the severity of clinical and radiographic CIA. In established CIA, oral 2ME2 reduced disease severity compared with vehicle-treated controls.

## 5. Cytokines Show Angiogenic Activity

An in vitro investigation has reported angiogenic activity with IL-6 plus soluble IL-6 receptor (sIL-6R) in RA FLS co-cultured with human umbilical vein endothelial cells (HUVECs) [16]. Interestingly, whereas IL-6/sIL-6R complex induced tubule formation and augmented VEGF production in the co-culture system, IL-6 alone had no such effects. IL-6/sIL-6R-induced tubule formation was abolished by the addition of either anti-IL-6R or anti-VEGF antibody. Unlike IL-6/sIL-6R, TNF-α did not induce tubule formation; instead, TNF-α reduced the CD31-positive area compared with RA FLS co-cultured with HUVECs without cytokine augmentation (control).

IL-17A has been found to induce human dermal endothelial cell (HDEC) tube-like structures and extracellular matrix (ECM) invasion, and significantly increase the secretion of chemokines (growth-related oncogene-alpha (GRO-α) and monocyte chemotactic protein-1 (MCP-1)) from RASFs [17]. The same researchers also reported IL-17A induced migration of RASFs, HDECs, and mononuclear cells, which was blocked by anti-GRO-α or anti-MCP-1 antibodies. Interestingly, the studies showed that IL-17A differentially regulated αvβ3 and αvβ1 integrin expression, and induced cytoskeletal rearrangement and upregulation of active Rac1, key markers in angiogenic vascular morphology and cell migration.

It may be worthwhile targeting IL-18 or its signaling intermediaries in RA, according to a study confirming IL-18-induced angiogenesis in RA synovial tissue engrafted in severe combined immune-deficient (SCID) mice [18]. In that study, IL-18-induced human microvascular EC (HMVEC) chemotaxis, tube formation, and angiogenesis in Matrigel plugs was blocked by Src and c-Jun N-terminal kinase (JNK) inhibitors, whereas inhibitors of Janus kinase 2 (JAK2), p38, MEK, phosphatidylinositol-3-kinase (PI3K) and neutralizing antibodies to VEGF or stromal-derived factor-1α did not alter IL-18-induced HMVEC migration. The study researchers also report that IL-18 induced Src and JNK phosphorylation in HMVECs.

An in vitro investigation into the mechanism whereby IL-18 contributes to excessive angiogenesis has shown that IL-18 acts synergistically with IL-10 to amplify the production of M2 macrophage (Mφ)-derived mediators like osteopontin (OPN) and thrombin, yielding the thrombin-cleaved form of OPN, which acts through integrins α4/α9 and augments M2 polarization of Mφ with increasing surface CD163 expression in association with morphological alteration [19]. Furthermore, CD163 appears to mediate the direct cell-cell interaction between Mφs and ECs during angiogenesis.

Other research indicates that IL-11 appears to represent a novel connection between RA joint fibroblasts and ECs, enhancing synovial fibroblast infiltration and further advancing disease severity by increasing the invasion of blood vessels into the RA pannus [20]. Blocking IL-11 impaired RASF capacity to elicit EC transmigration and tube formation.

### TLR2 May Amplify the Effects of Serum Amyloid A

Serum amyloid A (A-SAA), an acute-phase protein with cytokine-like properties, promotes cell migrational mechanisms and angiogenesis critical to RA pathogenesis [21]. As that paper observes, the fact that other research has reported localization of TLR2 expression to the RA synovial lining layer and synovial macrophages is consistent with the localized expression of A-SAA, so TLR2 may play a role in the A-SAA-mediated response in RA.

Resistin may be an appropriate target in RA. Resistin promotes endothelial progenitor cell (EPC) homing into the synovium during RA angiogenesis via a signal transduction pathway involving VEGF expression in primary EPCs [22]. Others suggest that it may be useful to inhibit leptin in RA disease, as leptin promotes RA FLS migration by increasing reactive oxygen species (ROS) expression [23]. Leptin is also capable of enhancing HUVEC tube formation in a ROS/HIF-1α-dependent manner, and promoting production of VEGF and IL-6 in RA FLS. It is possible to downregulate leptin-induced ROS production with the use of TNF, IL-6 and IL-1β antagonists, and thus attenuate RA FLS migration and HUVEC tube formation.

IL-1β has been found to play an important role in chondrocyte angiogenesis. IL-1β stimulation of chondrogenic ATDC5 cells increased FGF-2 expression and promoted EPC tube formation and migration [24]. The same research group reports finding that FGF-2-neutralizing antibody abolished ATDC5-conditional medium-mediated angiogenesis in vitro, as well as its angiogenic effects in the chick chorioallantoic membrane (CAM) assay and Matrigel plug nude mice model in vivo.

## 6. Targeting Stromal Cells and Vascular Responses

Some evidence indicates that targeting stromal cell-derived pro-angiogenic factors and HIF transcriptional responses can reduce the contribution of fibroblasts to the chronic inflammatory response [25,26]. In one study, chronically inflamed synovial tissue from patients with RA or OA significantly enhanced myeloid cell infiltration and angiogenesis in immune-deficient mice, which was associated with increased constitutive and hypoxia-induced VEGF expression in inflammatory fibroblasts compared with healthy fibroblasts [25]. A single intra-peritoneal (IP) injection of a VEGF antagonist (bevacizumab 5 mg/kg) at the time of Matrigel injection significantly inhibited angiogenesis and myeloid cell infiltration. Similar effects were seen in mice treated with daily IP injections of a CXCL12/CXCR4 antagonist (bicyclam AMD3100 at a dose of 300 µg). Targeting HIF-1α expression by lentiviral siRNA transduction of RA fibroblasts reduced both HIF-1α accumulation and significantly reduced angiogenesis in RA fibroblasts.

Hypoxia increases the angiogenic drive of RA cells, by upregulating MMPs responsible for collagen breakdown (MMP-2, MMP-8, and MMP-9), at both mRNA and protein levels [27]. These researchers also describe how hypoxia significantly increases RA fibroblast migration across collagen and is dependent on MMP activity in an in vitro angiogenesis assay. They document increased expression of angiogenic stimuli, such as VEGF, and VEGF/placental growth factor heterodimer.

In rats with adjuvant arthritis, significantly up-regulated levels of VEGF, HIF-1α, and CD34 expression have been observed in synovial tissue [28]. Significant, positive correlations were observed between VEGF mRNA and extent of paw swelling, between HIF-1α protein and the arthritis index, while VEGF mRNA and HIF-1α protein were positively correlated with CD34. Clearly, hypoxia is closely linked to angiogenesis and inflammation in RA; angiogenesis blockade is a worthwhile therapeutic concept.

### Possibilities of Stem Cell Therapy and GZMB Gene Silencing

Exogenously administered mesenchymal stromal cells (MSCs) inhibit dendritic cell maturation, promote macrophage polarization towards an anti-inflammatory phenotype and activate regulatory T cells, thereby lowering inflammation and preventing joint damage [26]. Proof-of-concept clinical studies have shown that allogeneic MSC therapy has a satisfactory safety profile and promising efficacy in the management of RA. More data are needed from larger, multicenter studies. Interestingly, when researchers explored the underlying mechanisms of human bone marrow-derived MSCs administered to mice with collagen antibody-induced arthritis (CAIA), they found that the curative effects of MSCs appear to depend on their migration into inflamed tissue, where they directly induce the differentiation of CD4^+^ T cells into regulatory T cells, and thus suppress inflammation [29]. Such evidence supports the systemic administration of MSCs in the setting of RA.

Investigations suggest that the serine proteinase granzyme B (GZMB) may be a useful prognostic marker in early RA. In CIA rats, silencing of the *GZMB* gene helped to maintain body weight increases, reduce the degree of ankle swelling, as well as relieve RA synovial tissue hyperplasia and articular cartilage tissue injury [30]. *GZMB* gene silencing decreased inflammatory cytokine levels and also Bcl-2, cyclin D1, VEGF and basic fibroblast growth factor (bFGF) expression, while simultaneously increasing mRNA and protein levels of caspase 3.

Recent research indicates a novel role for galectin-9 (Gal-9), a mammalian lectin secreted by ECs that is highly expressed in RASFs and synovial tissues. In a series of in vitro and in vivo investigations, Gal-9 medium significantly increased HMVEC migration and tube formation on Matrigel, as well as in vivo angiogenesis, via the ERK1/2, p38, and JNK pathways [31]. Gal-9 medium also induced monocyte migration and acute inflammation when injected into C57BL/6 mouse knees, indicating a proinflammatory role for Gal-9.

## 7. Characterizing the Expression and Function of Chemokine Receptors in RA

CCR7 signaling is essential in the pathogenesis of RA [32]; the development of lymphoid neogenesis in RA depends on the homeostatic chemokine receptors CXCR5 and CCR7 [33]. An essential role has also been identified for CCL28, a CCR10 ligand, in RA pathogenesis. The production of CCL28 from joint myeloid and ECs strongly promotes angiogenesis in EPCs and it is now known that both CCL28 and CCR10 are involved in RASF-mediated EPC chemotaxis [34]. The same researchers have also demonstrated that CCL28 can directly mediate neovascularization by attracting CCR10^+^ ECs. The CCL28/CCR10 cascade is a potential therapeutic target for RA. The CCL19 and CCL21 pathways also play important roles in RA angiogenesis [35].

## 8. Targeting the MMP Family

Inhibition of CD147 may reduce angiogenesis in RA. CD147, also known as extracellular matrix metalloproteinase inducer (EMMPRIN), is highly expressed in RA synovial tissue and triggers human synoviocytes to produce MMPs. Investigations have shown that CD147 expression is significantly and positively correlated with VEGF and HIF-1α levels, as well as with vascular density, in RA synovium [36]. When those researchers transfected RA FLS with the CD147-specific small interfering RNA (siCD147) or specific antibodies for CD147, VEGF, and HIF-1α expression was significantly decreased. In vivo findings in SCID-HuRAg mice were consistent with the in vitro findings, with both systems showing that CD147 up-regulation on RA FLS induces the up-regulation of VEGF and HIF-1α, which may further augment angiogenesis. 

Another research group has suggested that the PI3K/Akt pathway may underlie CD147-induced upregulation of VEGF in U937-derived foam cells [37]. When the cell culture was transfected with CD147 stealth siRNA, the extent to which VEGF production was reduced depended on the inhibition efficiency of CD147 siRNAs. The addition of signaling pathway inhibitors LY294002, SP600125, SB203580, and U0126 to cultures revealed that LY294002 dose-dependently inhibited the expression of VEGF. Phospho-Akt was also reduced in both the LY294002 and siRNA groups.

An investigation into the role of the cell surface metalloproteinase ADAM-10 (a disintegrin and metalloprotease 10) in RA angiogenesis has reported high levels of ADAM-10 in ECs and lining cells within RA synovial tissue compared with cells from OA and normal synovial tissue [38]. After incubation for 24 h with proinflammatory mediators phorbol myristate acetate (PMA), lipopolysaccharide (LPS), IL-17, IL-1β, or TNF-α, the researchers observed significantly elevated ADAM-10 expression at both the protein and messenger RNA levels in HMVECs and RASFs as compared with unstimulated cells. In addition, EC tube formation and migration was lower in ADAM-10 siRNA-treated HMVECs when compared with control siRNA-treated HMVECs. When untreated HMVECs, ADAM-10 siRNA-treated HMVECs, and control siRNA-treated HMVECs were co-cultured with RASFs, EC tube formation was reduced in ADAM-10 siRNA-treated HMVECs compared with control siRNA-treated HMVECs. As the researchers suggest, ADAM-10 appears to be a potential therapeutic target in RA.

## 9. Chinese Herbal Preparations

Scopolin isolated from *Erycibe obtusifolia* Benth stems has long been used in traditional Chinese medicine for the treatment of RA. In an adjuvant-induced arthritis (AIA) rat model, animals treated with high doses of scopolin (100 mg/kg) had higher mean body weights, near-normal histology of joint architecture and significantly reduced angiogenesis in synovial tissue compared with untreated controls [39]. The study researchers suggest that scopolin could potentially be used to treat angiogenesis-related disorders and serve as a structural base for screening more potent synthetic analogs.

Another traditional Chinese herbal compound, *Celastrus aculeatus* Merr. (*Celastrus*), has been used in China for centuries to treat rheumatoid disease. Celastraceae plants contain pristimerin, a triterpenoid quinone methide isolated from *Maytenus heterophylla*, a Kenyan medicinal plant. It appears that pristimerin has anti-angiogenic potential in RA. In AIA rats, pristimerin significantly decreased vessel density in synovial membrane tissues of inflamed joints and reduced the expression of pro-angiogenic factors TNF-α, angiopoietin 1 (Ang-1), and MMP-9 in sera [40]. Pristimerin also reduced synovial membrane expression of VEGF and phosphorylated VEGF receptor 2 (pVEGFR2), suppressed capillary sprouting in the rat aortic ring and inhibited migration of VEGF-induced RA-human fibroblast-like synoviocytes (HFLS) in vitro. Furthermore, pristimerin inhibited VEGF-induced proliferation, migration and tube formation by HUVECs, blocked the auto phosphorylation of VEGF-induced VEGFR2 and downregulated VEGFR2-mediated activation of PI3K, Akt, mTOR, ERK1/2, JNK, and p38.

## 10. Other Potentially Targetable Factors That Participate in RA Angiogenesis

Some evidence suggests that benzophenone analogs could have a role in ameliorating RA. Some researchers have demonstrated that commencing treatment with the synthetic benzophenone analog 2-benzoyl-phenoxy acetamide (BP-1) after the onset of disease in an AIA rat model reduced the arthritic score, paw volume and edema, the degree of inflammation and redness, as well as bone erosion, compared with untreated rats [41]. The researchers report that VEGF expression was clearly down-regulated in hypoxic ECs and AIA rats administered BP-1. Nuclear translocation of HIF-1α was also inhibited in synovium tissue after BP-1 treatment, which subsequently suppressed transcription of the *VEGF* gene.

Evidence suggests that proprotein convertase subtilisin/kexin type 6 (PCSK6) may serve as an important therapeutic target in RA. Stimulation with recombinant human (rh)PCSK6 of cultured RASFs from RA patients significantly increased RASF cell invasion, migration, and proliferation, which was influenced through both reduced cell cycle arrest and reduced apoptosis [42]. rhPCSK6 also stimulated RASFs to secrete IL-1α, IL-1β, and IL-6, and altered gene expression patterns involved in angiogenesis, hypoxia, proliferation, and inflammation. The signaling pathways involved in these cellular effects included the NF-κB, signal transducer and activator of transcription 3 (STAT3) and ERK1/2 pathways.

Other evidence points to the involvement of lysyl oxidase (LOX) in the promotion of synovial hyperplasia and angiogenesis in CIA rats [43]. In this study, the researchers identified higher amounts of rough synovial membranes, higher microvascular density in those membranes and more synovial cell layers in CIA rats compared with saline-treated controls. The CIA rats also exhibited higher LOX enzymatic activity and higher MMP-2 and MMP-9 expression levels compared with controls. Injection of CIA rats with the LOX inhibitor β-aminopropionitrile inhibited paw swelling and decreased the arthritis index, microvascular density in the synovial membranes and MMP-2 and MMP-9 expression levels. Notably, LOX expression levels in the synovial membranes were positively associated with microvascular density, as well as with levels of MMP-2 and MMP-9 expression.

Calreticulin, a multi-functional endoplasmic reticulum protein, has been found to promote RA-related angiogenesis via the activating nitric oxide (NO) signaling pathway [44]. Calreticulin concentrations were significantly higher in serum samples from RA patients than in serum samples from OA patients and healthy controls, and significantly higher in synovial fluid from RA patients than that OA patients. Calreticulin increased NO production and endothelial nitric oxide synthase (eNOS) phosphorylation in HUVECs, and promoted their proliferation, migration and tube formation. The effects of calreticulin on the proliferation, migration and morphological differentiation of HUVECs were significantly inhibited by l-NAME, a specific eNOS inhibitor.

### Targeting Proinflammatory YKL-40, Cyr61/CCN1, Axna2, and Axna2R

The proinflammatory protein YKL-40, also known as human cartilage glycoprotein-39 or chitinase-3-like-1, reportedly stimulates IL-18 production in osteoblasts and facilitates EPC angiogenesis [45]. The study researchers found that this process occurs through the suppression of miR-590-3p via the focal adhesion kinase (FAK)/PI3K/Akt signaling pathway. In vivo models of angiogenesis (CAM and Matrigel plug models) confirmed that inhibition of YKL-40 reduced angiogenesis.

Recent observations report that the proinflammatory cytokine cysteine-rich 61 (Cyr61 or CCN1), a secreted protein from the CCN family, induces VEGF expression in osteoblasts and increases EPC angiogenesis in RA [46]. The evidence reveals two major mechanisms through which CCN1 stimulates EPC-dependent angiogenesis. CCN1 inhibits the microRNA miR-126, a potent VEGF inhibitor, inhibitor of angiogenesis, and tumour suppressor, via the protein kinase C-alpha (PKC-α) signaling pathway. Thus, inhibition of miR-126 indirectly stimulates angiogenesis. CCN1 also directly increases VEGF expression in and production by osteoblasts. In vitro and in vivo investigations demonstrated that angiogenesis was inhibited by CCN1 knockdown. In CIA mice injected with lentiviral vectors expressing CCN1 short hairpin RNA (Lenti-CCN1), hind paw swelling was significantly ameliorated compared with that of mice in the control group. Lenti-shCCN1 treatment was also associated with markedly lower numbers of cells positive for CCN1, EPC markers (CD34 and CD133), and vessel markers (CD31, CD144, Endomucin, and VEGF), as well as less cartilage erosion, compared with untreated CIA mice.

Annexin A2 (Axna2) and its receptor (Axna2R) are upregulated in patients with RA compared with patients with OA and healthy controls [47]. Moreover, in CIA mice, exogenous Axna2 promotes the development of arthritis, by aggravating the disease process and joint damage. Suppressing the effects of Axna2 could therefore ameliorate RA pathogenesis.

## 11. Osteoarthritis

**Figure 2:**
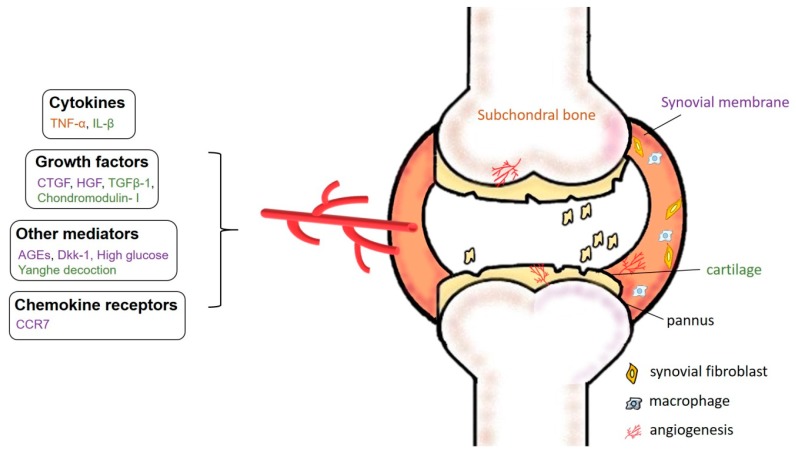
Specific mechanisms underlying angiogenesis in OA. Chronic, low-grade inflammation in OA is driven by increased expression of pro-angiogenic factors including chemokine receptors, cytokines, growth factors, and other mediators such as advanced glycation end-products (AGEs) and Dickkopf-1 (Dkk-1) entering the synovial fluid, enabling them to erode cartilage and subchondral bone.

Inflammation in OA differs from that in RA. OA pathogenesis is characterized by chronic, low-grade inflammation within the synovial lining [48], whereas patients with RA manifest with persistent, high-grade systemic inflammation [49]. In both diseases, it is essential to halt the inflammatory process, which prevents proper repair by bone, stromal, and/or cartilage cells (see Figure 2). American College of Rheumatology (ACR), American Academy of Orthopaedic Surgeons (AAOS), and Osteoarthritis Research Society International (ORSI) guidelines recommend the use of various symptom-modifying agents for the treatment of OA, which mainly fall into five categories: acetaminophen; opioid analgesics; nonsteroidal anti-inflammatory drugs (NSAIDs); intra-articular injections (corticosteroids and hyaluronic acid); and serotonin-norepinephrine reuptake inhibitors (duloxetine) [2]. Although anti-inflammatory modalities have shown promise in in vitro and preclinical models of OA, there are currently no established United States Food and Drug Administration (US FDA)/European Medicines Agency (EMA)-approved therapies that inhibit the low-grade inflammation in this disease [2,3]. As discussed below, the emerging evidence suggests several promising avenues for pharmacologic therapies that might eventually be used to prevent or slow OA disease progression in patients.

## 12. Angiogenesis in the OA Synovium

The role of the inflammatory mediator connective tissue growth factor (CTGF/CCN2) has been investigated in VEGF production and angiogenesis in OA synovial fibroblasts (OASFs) [50]. It appears that CTGF activates the PI3K, Akt, ERK, and NF-κB/ELK1 pathways, leading to the up-regulation of miR-210, contributing to the inhibition of GPD1L expression and prolyl hydroxylase 2 activity, promoting HIF-1α-dependent VEGF expression and angiogenesis in human SFs (see Figure 2).

The discovery that CCR7 is functionally expressed on FLS of patients with RA and OA has revealed that this process enhances VEGF secretion in both diseases [32]. Other researchers have found that hepatocyte growth factor induces concentration- and time-dependent increases in VEGF-A expression in OASFs and that this enhancement involves the activation of the c-Met/PI3K/Akt and mTORC1 pathways [51].

Implantation of inflammatory SFs from patients with chronic arthritis (RA or OA) into immune-deficient mice has been found to enhance myeloid cell recruitment and angiogenesis [25]; these proangiogenic factors correlate with increasing levels of VEGF expression. VEGF and CXCL12 antagonists significantly reduced myeloid cell infiltration and angiogenesis.

## 13. The Importance of Targeting AGE-Induced Inflammatory Responses

Interference with the activity of the Wnt inhibitor Dickkopf-1 (Dkk-1) has been shown to reduce the expression of angiogenic factors and proteinases, as well as ameliorate synovial vascularity and cartilage injury in an animal model of OA knee joints [52], while insight into the signaling pathway of advanced glycation end-products (AGEs) has led to the understanding that AGEs induce the expression of COX-2 and the production of prostaglandin E2 (PGE2), IL-6 and MMP-13 in human OA synoviocytes [53]. Neutralizing antibody for the receptor for AGEs (RAGE) effectively reversed the AGE-induced inflammatory responses and VEGF production in human synoviocytes, indicating that RAGE plays an important role in the activation of synoviocytes and thereby encourages OA progression. Investigations have highlighted the link between IL-1β and chondrocyte angiogenesis in arthritis. As noted in the RA section above, IL-1β stimulation of chondrogenic ATDC5 cells induces FGF-2 expression and promotes EPC tube formation and migration [24]. FGF-2-neutralizing antibody abolishes ATDC5-conditional medium-mediated angiogenesis in vitro, as well as its angiogenic effects in the chick chorioallantoic membrane (CAM) assay, Matrigel plug nude mice model, and CIA mouse model. In these studies, IL-1β was found to induce FGF-2 expression via IL-1RI, ROS generation, AMP-activated protein kinase (AMPK), the AMPK-dependent p38 pathway, and the NF-κB pathway.

Other researchers have demonstrated that high glucose induces VEGF production in OASFs [54]. They report that high glucose generates increases in ROS production and induces concentration- and time-dependent increases in VEGF expression. This increase in VEGF production is inhibited by pretreating OASFs with NADPH oxidase inhibitors (APO or DPI), a ROS scavenger (NAC), a PI3K inhibitor (Ly294002 or wortmannin), an Akt inhibitor, or AP-1 inhibitor (curcumin or tanshinone IIA). High glucose treatment also increases PI3K and Akt activation and increases the accumulation of phosphorylated c-Jun in the nucleus, AP-1-luciferase activity, and c-Jun binding to the AP-1 element on the VEGF promoter.

## 14. Targeting OA Cartilage

Targeting the transforming growth factor β1 (TGF-β1) or relevant receptors may help to prevent or lessen angiogenic activity in OA. One group of researchers has reported that TGF-β1 treatment of human chondrocytes cultured in vitro significantly upregulates genes involved in chondrocyte hypertrophy and blood vessel development [55]. Another potential strategy for attenuating angiogenic activity in OA cartilage is to target chondromodulin-I (ChM-I) expression [56]. Investigations have shown that in mildly degenerated human OA cartilage, ChM-I expression is significantly decreased in the extracellular matrix (ECM) of the superficial zone and in the cytoplasm of the superficial and middle zones compared with normal cartilage (*p* < 0.05). In moderately degenerated cartilage, ChM-I protein expression is reduced in the ECM of all zones of articular cartilage, but the immunostaining intensity in the cytoplasm is increased. In severely degenerated cartilage, ChM-I expression is detected primarily in the cytoplasm of the cluster-forming chondrocytes. The density of vascular channels correlates with levels of ChM-I expression in cartilage ECM. The findings suggest that loss of ChM-I may promote angiogenesis in OA cartilage.

## 15. Subchondral Bone and Articular Cartilage

Some researchers suggest that it may be possible to prevent or reduce joint pathology and pain symptoms in OA by reducing angiogenesis and nerve formation from the subchondral bone into articular cartilage [57]. Their data demonstrated associations between neurovascular growth and expression of proangiogenic factors VEGF, nerve growth factor (NGF) and platelet-derived growth factor (PDGF) at the osteochondral junction. Other researchers suggest that angiogenesis and associated sensory nerve growth in human menisci may be a potential source of pain in knee OA [58]. They found that this increased vascular penetration and nerve growth expression enhanced inflammation and tissue damage, driving OA pathogenesis. OA has also been associated with deficient fluid clearance. Synovia from patients with knee OA is associated with lower lymphatic vessel density (LVD) and lower lymphatic EC fractional areas than synovia from non-arthritic control knees [57]. In the OA cohort, low LVD was associated with clinically detectable lesions. The study researchers hypothesized that impaired SF drainage, due to reduced LVD, may contribute to effusion in OA.

Using lenalidomide to inhibit the activity of TNF-α and leucine-rich-alpha-2-glycoprotein 1 (LRG1) appears to attenuate OA progression [59]. LRG1 expression was upregulated in the subchondral bone and articular cartilage in a mouse OA model (anterior cruciate ligament transection [ACLT] mice) and was associated with angiogenesis. The researchers also found that TNF-α stimulated LRG1 expression in HUVECs and that this effect was inhibited through p38 and NF-κB signaling. The injection of lenalidomide reduced the number of nestin-positive MSCs in the subchondral bone of ACLT mice compared with sham-operated controls. Lenalidomide also reduced the number of osterix-positive osteoprogenitors. Lenalidomide not only attenuated the pathological changes of subchondral bone but also alleviated the degeneration of articular cartilage compared with vehicle-treated mice. Of all surgically-induced OA models, the ACLT model is currently the most commonly used [60]. Other commonly used models include meniscectomy (partial and total), medial meniscal tear, and ovariectomy. Using aseptic techniques to surgically induce OA in animals yields highly reproducible results that progress rapidly. The ACLT model imitates articular cartilage degradation after ACL injury. As the OA lesions in this model develop more slowly than after meniscectomy, ACLT is useful in pharmaceutical investigations.

A Chinese medicinal formulation, Yanghe Decoction, has indicated in preclinical investigations that it may protect articular cartilage in the early stage of OA [61]. The formula contained *Rehmannia glutinosa* (30 g), cinnamon (3 g), ephedra (2 g), antler gum (9 g), white mustard seed (6 g), ginger charcoal (2 g) and licorice (3 g). A rabbit model of OA was established using New Zealand white rabbits randomly allocated to one of three groups: normal healthy controls, untreated OA, or OA treated with Yanghe Decoction for 14 days administered at the end of the study; all animals were sacrificed at 8 weeks. Examination of tibia articular cartilage revealed significant between-group differences for Mankin scores (1.25 vs. 6.25 and 3.22 in the controls, untreated and treated animals, respectively; *p* < 0.01 for both comparisons). Moreover, IHC (immunohistochemistry) staining revealed a significantly higher level of VEGF expression in the untreated OA group compared with controls (1.49 vs. 0.83; *p* < 0.01) and the Yanghe Decoction group (1.05; *p* < 0.05).

## 16. Summary and Future Directions

The evidence discussed in this Review underlines the essential role played by angiogenesis in RA and OA in articular cartilage. Angiogenic activity initiates and perpetuates both arthropathies, contributing to inflammation, joint damage and pain. Notably, the effects of angiogenesis have been confirmed as extending beyond the RA synovium to include RA osteoblasts and also OA subchondral bone and articular cartilage. For example, in preclinical investigations, elevated levels of the pro-inflammatory cytokine TNF-α-induced LRG1 expression during OA progression [32]. Using lenalidomide to inhibit TNF-α successfully reduced TNF-α-induced LRG1 secretion and attenuated degeneration of OA articular cartilage. Interestingly, besides demonstrating angiogenic and anti-inflammatory effects, lenalidomide has also shown antitumor activity in clinical trials for the treatment of multiple myeloma and colorectal cancer, highlighting the importance of angiogenesis as a therapeutic target [62,63,64,65,66]. Other researchers have reported that IL-1β induces FGF-2 expression and promotes EPC angiogenesis in chondrocytes, then subsequently promotes EPC migration and tube formation [24]. Similarly, VEGF production in osteoblasts and EPC angiogenesis is promoted by CCN1, which has been found to inhibit levels of miR-126 expression in RA [46]. Another facet of angiogenesis is the activity of MSCs, which may have potential in RA and OA management [26,67]. Similarly, platelet-rich plasma (PRP) shows potential in arthritis. More high-quality clinical evidence is needed to determine the effectiveness of such therapy in the management of articular cartilage pathology [68] and to confirm its promising data in experimental studies [69]. All in all, the expression of chemokines and cytokines in synovial fluid is the most important element to consider in RA and OA angiogenesis—these molecules control the disease and the more that we learn about the complex process involved in their networks of anti- and pro-inflammatory interactions, the closer we surely edge towards the day when we can arrest these chronic diseases at their earliest stages.

## Figures and Tables

**Table 1:** Literature consulted for the RA section of this Review.

Stimulation	Target Factors		Effects in Tissue	Known Pathways	References
*Toll-like receptors*					
Toll-like receptor 3	VEGF, IL-8	↑	Synovium	NF-κB	[11]
Toll-like receptor 2	Ang2/Tie2	↑	HMVEC	Ang2/Tie2	[12]
*Cytokines*					
Resistin	VEGF	↑	EPC	PKC AMPK/miR-206	[22]
Leptin	VEGF, IL-8	↑	Synovium	ROS/HIF-1	[23]
IL-11	VEGF, IL-8	↑	Synovium	N/A	[20]
IL-18, IL-10	OPN	↑	M2 macrophage (Mφ)	N/A	[19]
Acute serum amyloid A			Synovium	N/A	[21]
IL-18	IL-18	↑	HMVEC	Src/JNK	[18]
IL-17A	IL-17A	↑	HDECs	N/A	[17]
IL-6/SL-IL-6R	VEGF	↑	Synovium	IL-6/SL-IL-6R	[16]
IL-1β	bFGF	↑	Cartilage	ROS/AMPK/p38/NF-κB	[24]
*Chemokine receptors*					
CCR7	VEGF	↑	Synovium	N/A	[32]
CCL28	CCR10	↑	Synovium and EPC	ERK1/2	[34]
CXCR5		↑	CIA model	N/A	[33]
*MMPs*					
CD147	VEGF, HIF-1α	↑	Synovium	PI3K/AKT/HIF-1α	[36]
ADAM-10	ADAM-10	↑	Synovium	N/A	[38]
*Chinese Herbs*					
Pristimerin	VEGF-A/VEGFR2	↓	Synovium	PI3K/AKT/mTOR and MAPK	[40]
Scopolin	IL-6, VEGF and FGF-2	↓	Synovium	N/A	[39]
*Growth factors*					
CCN1	VEGF-A	↑	Osteoblast	PKC/miR-126	[46]
VEGF	vasohibin-1	↓	Synovium	N/A	[13]
*Other mediators*					
YKL-40	IL-18	↑	Osteoblast	FAK/PI3K/AKT	[45]
Lysyl oxidase (LOX)	MMP-2, MMP-9	↑	Synovium	N/A	[43]
PCSK6	IL-1, IL-1, IL-6	↑	Synovium	NF-B	[42]
Galectin-9	Galectin-9	↑	HMVEC	N/A	[31]
*GZMB*	VEGF and bFGF	↑	CIA model	MEK/ERK	[30]
Annexin A2	VEGF, Ang-2, MMP-2	↑	HUVEC	HH signaling	[47]
HIF-1α	HIF-1, VEGF, CD34	↑	Synovium	HIF-1α	[28]
Hypoxia	VEGF and MMP-2, -8, -9	↑	Synovium	N/A	[27]
FK228 (inhibitor)	HIF-1α and VEGF	↓	Synovium	HIF-1α	[14]
2ME2 (inhibitor)	VEGF and bFGF	↓	Synovium	N/A	[15]
BP-1 (inhibitor)	HIF-1α and VEGF	↓	Synovium	HIF-1α	[41]

CCN1, CCN family member 1; VEGF, Vascular endothelial growth factor; VEGF-A, Vascular endothelial growth factor A; IL-11, Interleukin 11; IL-18, Interleukin 18; IL-10, Interleukin 10; OPN, Osteopontin; IL-17A, Interleukin 17A; IL-6, Interleukin 6; SL-IL-6R, Soluble interleukin 6 receptor; CCR7, C-C chemokine receptor type 7; CD147, Basigin; HIF-1α, Hypoxia-inducible factor 1-alpha; ADAM-10, A Disintegrin and metalloproteinase domain-containing protein 10; Ang2, Angiopoietin-2; Tie2, Angiopoietin-1 (Ang1) and Ang2 receptor tyrosine kinase; YKL-40, Chitinase-3-like protein 1; MMP-2, Matrix metalloproteinase 2; MMP-8, Matrix metalloproteinase 8; MMP-9, Matrix metalloproteinase 9; PCSK6, Proprotein convertase subtilisin/kexin type 6;IL-1α, Interleukin 1 alpha; IL-1β, Interleukin 1 beta; GZMB, Granzyme B; bFGF, Basic fibroblast growth factor; CD34, Cluster of differentiation 34; N/A, Not appropriate.

**Table 2:** Literature consulted for the OA section of this Review.

Stimulation	Target Factors		Effect in Tissue	Known Pathways	References
*Growth factors*					
CTGF	VEGF-A	↑	Synovium	PI3K/AKT/ERK and NF-B/ELK1	[50]
HGF	VEGF-A	↑	Synovium	c-Met/PI3K/Akt and mTORC1	[51]
TGF-β1	VEGF-A	↑	Cartilage	N/A	[55]
Chondromodulin-I	Chondromodulin-I	↑	Cartilage		[56]
*Chemokine receptors*					
CCR7	VEGF	↑	Synovium	N/A	[32]
*Other mediators*					
High glucose	VEGF-A	↑	Synovium	ROS, PI3K, Akt, c-Jun and AP-1	[54]
Dkk-1	Dkk-1	↑	Synovium	β-catenin– and ERK-dependent	[52]
AGEs	VEGF-A	↑	Synovium	RAGE-NF-κB pathway	[53]
*Cytokines*					
TNF-α	LRG1	↑	Subchondral bone	*p*38/ NF-κB	[59]
IL-1β	bFGF	↑	Cartilage	ROS/AMPK/p38/NF-κB	[24]
*Chinese Herbs*					
Yanghe Decoction	VEGF-A	↓	Cartilage	N/A	[61]

CTGF, Connective tissue growth factor; HGF, Hepatocyte growth factor; TGF-β1, Transforming growth factor beta-1; TNF-α, Tumor necrosis factor alpha; IL-1β, Interleukin 1 beta; Dkk-1, Dickkopf-related protein 1; AGEs, Advanced glycation end-products; N/A, not appropriate.
